# Supplementary material for: A multisensor high-temperature signaling framework for triggering daytime thermomorphogenesis in Arabidopsis
Source: Nat Commun. 2025 Jun 4;16:5197. doi: 10.1038/s41467-025-60498-7 (PMC12137955; doi:10.1038/s41467-025-60498-7)
Supplement: Supplementary file 1 — Supplementary Information [file 41467_2025_60498_MOESM1_ESM.pdf]

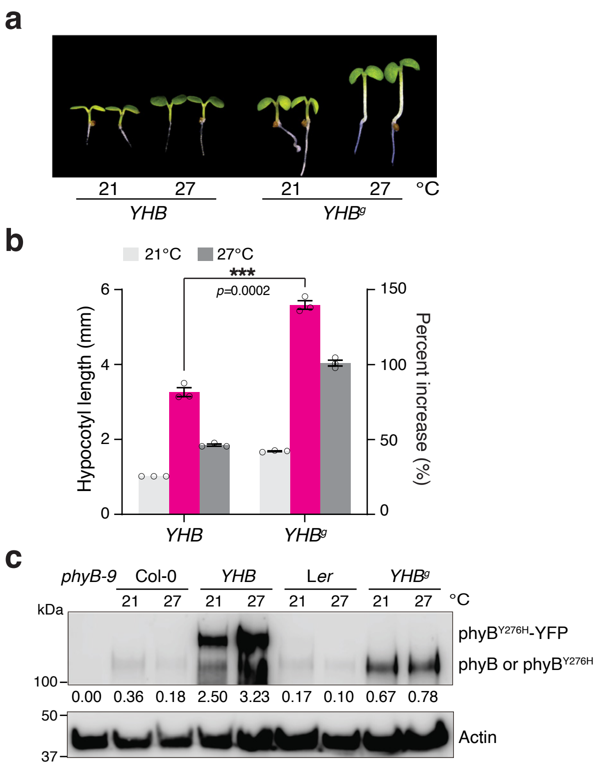

Supplementary Fig. 1. The discrepancy in the thermal responses of *YHB*^{g} and *YHB* may be attributable to different YHB levels. a Images of 4-d-old *YHB*^{g} and *YHB* seedlings grown under 50 μmol m^{-2} s^{-1} R light at 21 or 27 °C. **b** Hypocotyl length measurements of the *YHB*^{g} and *YHB* seedlings described in **a**. The light and dark gray bars represent hypocotyl lengths at 21 and 27 °C, respectively. The magenta bars represent the percent increase in hypocotyl length in the indicated lines between 21 and 27 °C. Error bars represent the s.e. (n = three biological replicates), and the centers of the error bars indicate the mean. A significant difference in the percent increase in hypocotyl length between the *YHB*^{g} and *YHB* was calculated using a two-tailed Student's t-test (*** $p < 0.001$). **c** Immunoblots showing the levels of phyB, phyB^{Y276H} or phyB^{Y276H}-YFP in Col-0, *YHB*, L*er*, and *YHB*^{g} seedlings grown under 50 μmol m^{-2} s^{-1} R light at either 21 or 27 °C. PhyB proteins were detected using anti-phyB antibodies, and actin was used as a loading control. The relative phyB levels normalized to the corresponding actin levels are shown. The underlying source data for the hypocotyl measurements in **b** and immunoblots in **c** are provided in the Source Data file.

Supplementary Table 1. List of primers used for qRT-PCR analysis.

Gene ID	Gene name	Forward primer	Reverse primer
AT1G13320	*PP2A*	TATCGGATGACGATTCTTCGTGCAG	GCTTGGTCGACTATCGGAATGAGAG
AT2G43010	*PIF4*	AACCAGATCATCTCCGACCGGTTT	TCCCGCCGGTGAACTAAATCTCAA
AT4G28720	*YUC8*	TGAAACAAAACAACCCACGA	TTGATTCGCTTTGGGTCTTC
AT1G04180	*YUC9*	GTCCCATTCGTTGTGGTCG	TTGCCACAGTGACGCTATGC
AT3G15540	*IAA19*	ATCGGTGTGGCCTTGAAAG	AACATCCCCCAAGGTACATC
