# Supplementary material for: A multisensor high-temperature signaling framework for triggering daytime thermomorphogenesis in Arabidopsis
Source: Nat Commun. 2025 Jun 4;16:5197. doi: 10.1038/s41467-025-60498-7 (PMC12137955; doi:10.1038/s41467-025-60498-7)
Supplement: Supplementary file 2 — Description of Additional Supplementary Information [file 41467_2025_60498_MOESM2_ESM.docx]

**Description of Additional Supplementary Files**

File Name: **Supplementary Data 1**

Description: ***Transcriptome analyses to determine genes co-regulated by dual thermosensors.*** RNA-seq analysis of 4-d-old hmr-5 and hmr-5pifq seedlings grown under 50 μmol m−2 s−1 R light at either 21 or 27 oC with or without sucrose.
